# The BRAIN-Q, a tool for assessing self-reported sport-related concussions for epidemiological studies

**DOI:** 10.4178/epih.e2021086

**Published:** 2021-10-19

**Authors:** Laura James, Madeline Davies, Saba Mian, Giulia Seghezzo, Elizabeth Williamson, Simon Kemp, Nigel Arden, Damien McElvenny, Neil Pearce, Valentina Gallo

**Affiliations:** 1 Centre for Primary Care and Public Health, Queen Mary University of London, London, UK; 2 University of Bath, Bath, UK; 3 London School of Hygiene & Tropical Medicine, London, UK; 4 Rugby Football Union, London, UK; 5 University of Oxford, Oxford, UK; 6 Institute of Occupational edicine, Edinburgh, UK; 7 Institute of Occupational Medicine and University of Manchester, Manchester, UK; 8 School of Public Health, Imperial College London, London, UK; 9 Department of Sustainable Health, Campus Fryslând, University of Groningen, Leeuwarden, Netherlands

**Keywords:** Questionnaire, Evaluation, Brain concussion, Sports medicine, Epidemiologic studies

## Abstract

**OBJECTIVES:**

The BRAIN-Q is a tool aimed at maximising the accuracy and minimising measurement error for retrospectively assessing concussions. This paper reports the agreement of the BRAIN-Q tool when compared to extant questionnaire questions, and its reproducibility when compared with its telephonic version (tBRAIN-Q).

**METHODS:**

The BRAIN-Q entails a 3-stage process: defining a concussion, creating a visual timeline with life events, and establishing detailed characteristics for each reported concussion. It was designed to be administered in-person by trained personnel, and was used in the BRAIN study. Its performance was compared with the MSK study, which previously collected a few questions in a broader self-administered questionnaire, and with the tBRAIN-Q Recall, its telephonic version.

**RESULTS:**

In total, 101 participants were included, of whom 9 were re-assessed with the tBRAIN-Q. The agreement of the BRAIN-Q with the muscle skeletal-questionnaire for rugby-related concussion was 86.7% (κ=0.6). Rugby-related concussion with loss of consciousness showed lower agreement (82.0%; κ=0.6). The comparison between the BRAIN-Q and the tBRAIN-Q showed good reproducibility.

**CONCLUSIONS:**

The BRAIN-Q is a relatively easy tool to administer in face-to-face assessments, and it showed optimal reproducibility. It includes a well-established definition of concussion, and is used to collect detailed information on each concussion, allowing for a number of subgroup analyses (e.g., by severity, age, or context). The BRAIN-Q is easily adaptable to other sporting settings.

## INTRODUCTION

Concussions occur as a result of trauma, and despite being recognised clinically for over a thousand years, have only been increasingly considered in sporting contexts in recent years [1]. Whilst there has been an awareness of post-concussion symptomology for many years, the prognosis for patients following concussion has received little attention until recent decades. The long-term effects of sport-related concussions are attracting increasing attention from the public and the scientific community due to the newly described chronic traumatic encephalopathy [2]. Additionally, in comparison with other sporting injuries, concussions are prominent across multiple sporting contexts, amenable to prevention and risk reduction efforts, and may predispose athletes to further risk of injury.

Increasing evidence suggests that exposure to sport-related concussions may increase the risk of neurodegenerative diseases later in life [3,4]. A recent systematic review on sport-related concussions and cognitive function concluded that the overall evidence points towards an association between sustaining a sport-related concussion and poorer cognitive function later in life in rugby, American football, and boxing [5]. Despite the mounting neuropathological evidence, and some initial studies in the field of rugby [6-8], several questions remain unanswered on the association linking the exposure to concussion to neuropathological and clinical prognoses.

In this context, when designing cross-sectional and case-control retrospective epidemiological studies, one of the main challenges is the assessment of self-reported exposure to previous concussions [5-8]. This is particularly true when assessing associations with poor cognitive function, as individuals suffering from cognitive decline may less accurately recall their exposure to concussions, potentially biasing the results. Adding to this challenge of retrospective exposure assessment, the definition of sport-related concussions has changed over time [9,10]. Anecdotal reports support the view that a few decades ago, loss of consciousness was required for a head impact to be defined as a concussion. This may have resulted in an underestimation of concussion in previous studies.

Careful consideration of these challenges has led the research team of the BRain health and healthy AgeINg in retired rugby union players (BRAIN) study [11] to develop a new tool aimed at maximising the accuracy and minimising measurement error when assessing self-reported concussions during face-to-face interviews: the BRAIN-Q tool. The aim of this paper is to report the agreement of the BRAIN-Q tool when compared to previously extant self-administered questionnaire questions, and to report its reproducibility when compared with its telephonic version (tBRAIN-Q).

## MATERIALS AND METHODS

**Figure 1.**
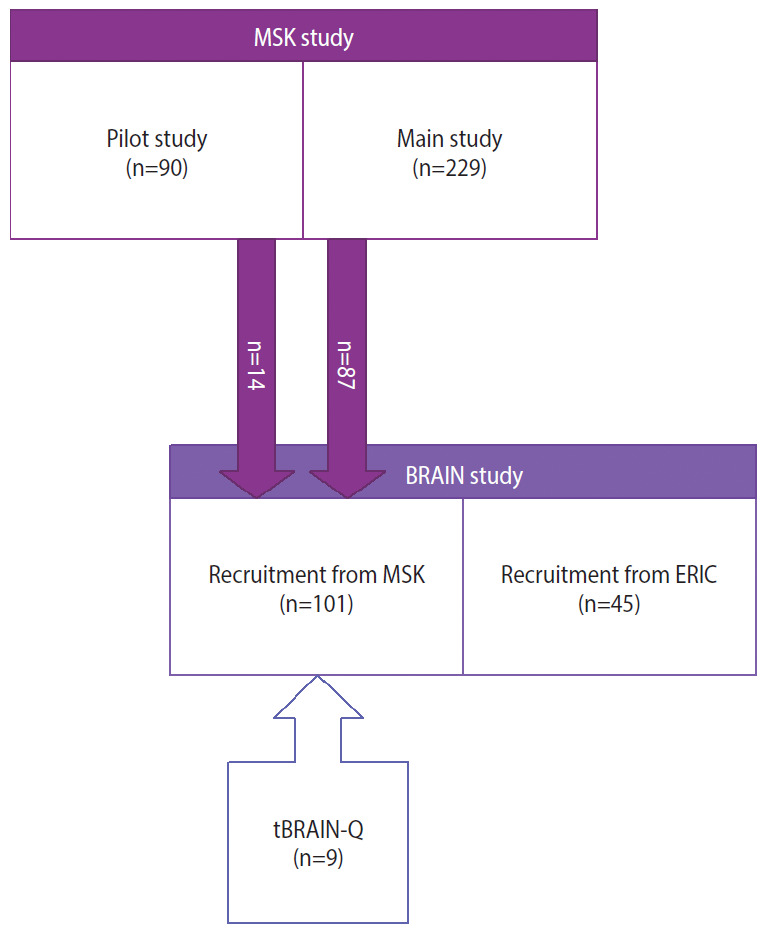
Flowchart depicting the sample for this study. 14 out of 90 participant assessed within the pilot MSK and 87 of the 229 assessed within the main Oxford and Cambridge University players and English international rugby players (MSK study) who were also recruited in the BRain health and healthy AgeINg in retired rugby union players (BRAIN) study formed the sample of the present study. ERIC, England Rugby Internationals Club; tBRAIN-Q, telephonic version of the BRAIN-Q.

The present analysis used information on sport-related concussions collected with 3 different tools, in 4 partially overlapping samples of male former elite rugby players, from 2 previous studies (Figure 1 and Table 1). All participants, for whom at least 2 different assessments carried out with 2 different tools were available for comparison, were included in this study. The studies and the tools used to assess concussions are described in detail below.

### The studies

#### The MSK study (pilot and main)

The Arthritis Research UK Centre for Sport Exercise and Osteoarthritis Rugby Epidemiology Questionnaire^©^ is a cross-sectional questionnaire-based study, carried out by the University of Oxford within the Centre for Sport, Exercise and Osteoarthritis Versus Arthritis, which assessed the general and musculoskeletal health of 319 former elite male Oxford and Cambridge University players and English international rugby players (‘MSK study’) [12]. The pilot study initially recruited former Oxford and Cambridge University rugby-playing participants using an online questionnaire (n=90), and then a modified questionnaire was produced and available postally or online, and distributed to both Oxbridge and former England international rugby-playing participants (n=229). Participants were recruited between August 2014 and February 2016. The median age of the players was 62.0 years (range, 24.2 to 95.0), with a mean playing exposure of 22.2±5.3 years, and 83.6% were amateur players.

#### BRAIN study

The BRAIN study is a cross-sectional study investigating the associations between self-reported concussion and cognitive function in retired elite male rugby players aged 50+ in England [11]. Participants were recruited to the BRAIN study between April 2017 and May 2019. The majority of the BRAIN study participants (n=101) were recruited from the earlier MSK study, and were included in the present analysis; given that a desirable sample size was not reached, a minority was recruited from a list of the England Rugby Internationals Club players [11]. Overall, the median age of the sample was 70.0 years (interquartile range [IQR], 61.0 to 77.0), they had a mean length of playing career of 15.8±5.4 years, their position of play was 45.0% backs and 55.0% forwards. Nine of these subjects were re-assessed with the telephonic version of the BRAIN-Q (the tBRAIN-Q tool) after the in-person assessment. For these 9 participants, the length of time between conducting the BRAIN-Q and then the tBRAIN-Q was at least 40 days.

For the purpose of the present analysis, subjects previously enrolled in the pilot and the main MSK study (pilot: n=14; main study: n=87), and subsequently enrolled into the BRAIN study—generating 2 non-overlapping samples—were included in the present analysis (n=101). In addition, 9 subjects assessed twice with the BRAIN-Q and tBRAIN-Q tools were analysed (Figure 1).

### The assessment tools

#### MSK study questionnaire (MSK-Q)

The concussion data collected in the MSK study took the format of a few questions within a broader self-administered questionnaire focussing on health, morbidity, musculoskeletal disorders, and joint pain (MSK-Q). In the pilot study, the data were collected using an online questionnaire, and in the main study, data were collected using a postal or online questionnaire.

A definition of concussion was outlined on the form for both pilot and main studies, before the rugby-related and non-rugby-related concussion questions were asked (Table 2). Following the definition, participants were asked: “Have you ever been dazed (‘dinged’) during a match?”, with possible answers including “yes”, “no”, or “don’t know”; and “Have you ever been unconscious (‘knocked out’) during a match?”, with responses of “yes” or “no”. In the main study only, total number of concussions (rugby-related and nonrugby-related) were collected with the question: “How many times have you been concussed? Please include all sporting and non-sporting concussions”, with answers of “concussed” and “don’t know” and the relative numeric answer or “don’t know”. In addition, players were asked questions on return to play, and if they had been seen by a neurologist, and other characteristics estimating concussion severity, which were not included in the present analysis. These questions were added to a self-administered questionnaire, and overall the time needed to complete this section by the respondent was estimated to be less than 5 minutes.

For the aim of this analysis, only the questions leading to the construction of numerical variables identifying the previous exposure to concussions and their numbers were included. Three dichotomous variables were created: rugby-related concussion, rugby-related concussion with loss of consciousness, and any concussion (rugby-related and non-rugby-related) (yes/no), allowing for respective missing values. In addition, 1 numerical variable was created, indicating the number of any concussions suffered. Differences in the size of the sample in which each variable was available were due to differences between the pilot and the main study questionnaire (Table 1).

#### BRAIN-Q tool

The BRAIN-Q is a concussion assessment tool that was developed for the BRAIN study and designed to be administered inperson by a trained research assistant. Careful consideration was given in designing the tool to elicit the most accurate assessment of concussion possible: the BRAIN-Q attempted to maximise the ability to obtain accurate concussion data by incorporating 3 core elements. Firstly, the BRAIN-Q gave a clear definition of concussion to the participant. The definition was developed using the National Institute of Health (NIH) concussion definition [13], and the language was simplified in order to make it accessible to a wider audience (panel 1). Participants in the BRAIN study were asked to read aloud the concussion definition before specifying the number of times they had been concussed, both during rugby and outside of rugby. Secondly, to assist the participant in recalling the number of sport-related and non-sport-related concussions he suffered during his lifetime, the BRAIN-Q offered a visual timeline. The timeline was derived on the basis of high-level questions about their playing career and life events which benchmarked some meaningful periods (e.g., school years, when they started playing at the varsity level, when they started playing at the professional level, and their play during their post-elite-level career). Each participant was asked to confirm their first self-reported number of concussions after using the timeline to record them. Lastly, for each self-reported concussion, the BRAIN-Q asked detailed questions on age, severity, loss of consciousness, together with some contextual information such as whether the concussion was sport-related or not. Information on severity included fracture of the skull or any other head bones, admission to hospital, or evaluation in the Accident and Emergency department without overnight admission. Information on the contextual factors included whether the concussion was experienced while playing/training for rugby, playing/training for another sport, motor vehicle accident, or other. The time needed to complete the BRAIN-Q test was estimated to be between 5 minutes and 10 minutes, depending on the length of rugby career and the number of concussions to be recorded. The full BRAIN-Q assessment tool is available as Supplementary Material 1.

For the purpose of this analysis, information from the BRAIN-Q tool was used to generate 3 dichotomous variables (rugby-related concussion, rugby-related concussion with loss of consciousness, and any concussion), and 1 discrete variable (number of any concussions suffered) (Table 1), which could be compared to the 4 generated MSK study variables.

#### The tBRAIN-Q Recall

The tBRAIN-Q Recall (telephonic version of the BRAIN-Q) was carried out without the aid of a timeline, and with participants who had already undertaken the BRAIN-Q assessment. The tBRAIN-Q Recall was administered in order to assess the BRAIN-Q’s repeatability. A subsample of 22 participants was randomly selected from the BRAIN study (independently from any characteristics of the concussion previously reported); of these, 10 agreed to repeat the BRAIN-Q assessment by phone (tBRAIN-Q), of whom only 9 had also provided data for the MSK-Q.

During the telephone assessment, the definition of concussion provided to the participant in the original face-to-face assessment was repeated to the participants. The information collected generated the same variables as the BRAIN-Q, displayed in Table 1.

### Data collection

In order to compare the data collected using the 2 tools, as mentioned previously, the concussion information from both studies was recoded, and 4 variables were derived that could be compared across the BRAIN and the MSK studies. These were 3 dichotomous variables (rugby-related concussion, rugby-related concussion with loss of consciousness, and any concussion), and 1 discrete variable (number of any concussions). These variables are available for the entire, or a subset of, the sample by design, and are shown in Table 1.

### Statistical analysis

Data available for the dichotomous variables were displayed in contingency tables, and the agreement of the MSK-Q in relation to the BRAIN-Q was calculated. A Bland-Altman plot, a graphical method used for evaluating the agreement between 2 quantitative measures [14], was produced for the discrete variable, and the limits of agreement were calculated. Concordance statistics were also calculated to assess the agreement between the BRAIN-Q and tBRAIN-Q.

### Ethics statement

All participants signed an informed or proxy consent form to participate in the study. The study received ethical approval from the University of Oxford Central University Research Ethics Committee (MSD-IDREC-C1-2014-020) and the study was approved by the Ethical Committee of the London School of Hygiene and Tropical Medicine (EC/11634) and further approved by the ethical committees of other participating institutions.

## RESULTS

A total of 101 participants who underwent the BRAIN-Q and also had concussion data recorded as part of the MSK-Q were included in the analysis. Of these, 9 participants were recalled to undertake the tBRAIN-Q. Only 3 dichotomous and 1 discrete variable could be compared between the 2 main studies: rugby-related concussion, rugby-related concussion with loss of consciousness, and any concussion (dichotomous); and the number of any concussions (Table 1).

The prevalence of rugby-related concussion using the BRAIN-Q was estimated to be 79.2% (80/101) in this sample; the same prevalence using the MSK-Q was estimated to be 81.6% (80/98). Similarly, the prevalence of rugby-related concussion with loss of consciousness was estimated to be 57.4% (58/101) in the BRAIN study and 53.0% (53/100) in the MSK study. The prevalence of any concussion using the BRAIN-Q was estimated to be 82.2% (83/101) in this sample; the same prevalence using the MSK-Q was estimated to be 79.1% (53/67).

Cross-tabulations with and agreement calculated for the dichotomous variables are shown in Table 3. For rugby-related concussion, the agreement between the two data collection methods was 86.7% (κ=0.6). The rugby-related concussion with loss of consciousness variable had a slightly lower agreement (82.0%; κ=0.6) than the previous variable. A similar analysis for any concussion showed an agreement between the 2 tools of 88.1%; the κ-value of 0.6 lies between moderate and substantial agreement.

**Figure 2.**
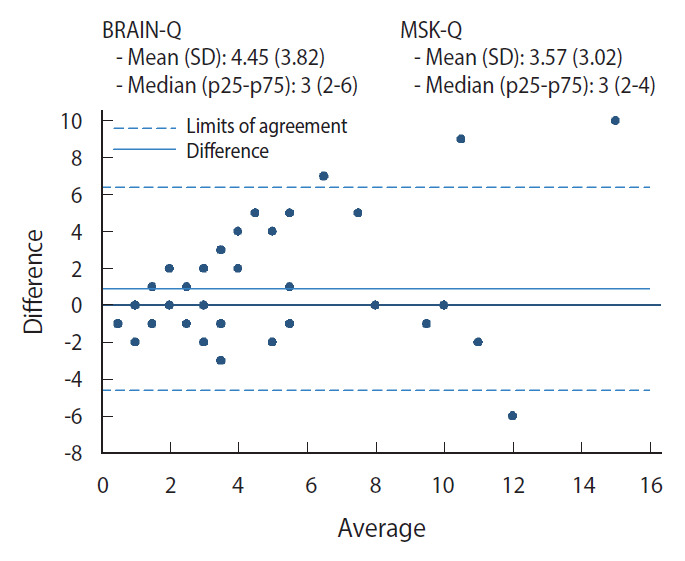
Bland-Altman plot – y-axis shows difference between the two concussion measures (BRAIN-Q and MSK study-main) and x-axis shows average number of any concussion among 53 participants. BRAIN, BRain health and healthy AgeINg in retired rugby union players; BRAIN-Q, BRAIN questionnaire tool; MSK study, Oxford and Cambridge University players and English international rugby players; MSK-Q, muscle skeletal questionnaire; SD, standard deviation; p, percentile.

The number of any concussions collected with the 2 methods and compared using a Bland-Altman plot shows the level of agreement between the methods (Figure 2): overall the BRAIN-Q recorded a slightly higher number of concussions (mean±standard deviation, 4.45±3.82) than the MSK-Q (3.57±3.02), with the differences between tools becoming higher with a higher number of self-reported concussions, specifically for more than 6 concussions (n=9).

The length of time between the BRAIN-Q assessment and follow-up phone call (tBRAIN-Q Recall) Ranged from 40 days to just over a year (368 days), with a median (IQR) of 121 days (IQR, 103 to 198). The comparison between the 2 sets of data (Table 4) shows that there was little change between the concussion data collected using the BRAIN-Q and the tBRAIN-Q Recall, with a high concordance reported for rugby-related and total number of concussions (ρ>0.9). This suggests that the BRAIN-Q is reproducible as a method for collecting concussion data. Since all participants underwent the BRAIN-Q before the tBRAIN-Q Recall, it is neither possible to assess the tBRAIN-Q independently nor to estimate the effect of the timeline on accuracy.

## DISCUSSION

This is the first study evaluating a tool designed specifically to recall past exposure to sport-related concussions. The BRAIN-Q is easy and relatively fast to administer, and it showed very good reproducibility.

The prevalence of rugby-related concussion measured with the BRAIN-Q tool is comparable to that measured with a simpler self-administered questionnaire. The agreement between the two tools was higher when any concussion was considered (88.1%), and slightly lower for concussions with loss of consciousness (82.0%), possibly suggesting that the interpretation of what constitutes losing consciousness is not always consistent. The analysis of the number of self-reported concussions for each individual suggested that the accuracy of reporting is reduced with an increasing number of concussions reported; the differences between the 2 methods was high for participants who reported 6 or more concussions.

The strengths of the BRAIN-Q are that it is relatively easy to administer in face-to-face assessments, showed optimal reproducibility, used a well-established definition of concussion, and collected detailed information on each concussion, enabling a number of subgroup analyses (e.g., by severity, age, or context). Moreover, it is easily adaptable to other sporting settings. The possible weaknesses of the tool are that it cannot completely account for potential misclassification bias of people with subclinical cognitive impairment recalling their exposure to concussion in a systematically different way compared to people without cognitive impairment. Additionally, the present data are somewhat limited by small numbers, in particular for selected comparisons (e.g., the number of rugby-related concussions), and possible selection bias of the tBRAIN-Q sample, which had a low response rate. The small sample size may also have affected the lack of certainty in the confidence intervals for specificity. The use of the NIH definition for concussion in the BRAIN-Q, ensures the robust capture of concussion data using an established and current definition; however, as has been mentioned, the definition of a concussion has evolved over recent years, and it is possible that we may have limited reporting by individuals with less common symptoms, or those which were not aligned with the NIH definition. The studies in which these tools were compared both involved male former rugby-playing populations. Females are at a higher risk of concussion, and implementing this tool for female samples, and in other sporting contexts, would support its generalisability outside of rugby and in more general settings.

The current results do not replicate observations of an increased number of concussion estimations after respondents received a definition of concussion among American footballers [15] and athletes from other sports [16]. This may be due to the fact that rugby players in England tend to be a highly educated group of people, the majority of whom have studied at the university level and generally show a good knowledge and understanding of the definition of concussion and its consequences. Additionally, this could have been affected by recent rugby-led concussion awareness campaigns, such as HEADCASE [17], reaching targeted playing, parental, and officiating audiences. However, it has been previously shown that player concussion knowledge may not prevent risk-taking behaviour, with 91% of Irish club and national rugby players being aware that they should not continue playing post-concussion, although 75% stating they would in an important game. O’Connell & Molloy [18] also found that 39% of players had tried to influence a medical assessment, showing how concussion knowledge may not always be reflected in safe behaviour.

In conclusion, the BRAIN-Q tool was found to improve the ability to identify rugby-related concussion in this sample, and showed good reproducibility when administered by phone. By using it in other studies, the consistency of results would be sensibly improved.

## Figures and Tables

**Table 1.** The 4 concussion variables used in the analysis and how they were derived from each data source

Derived variable	BRAIN-Q (n=101)	tBRAIN-Q Recall (n=9)	MSK study	
			Pilot (n=14)	Main (n=87)
Ever suffered a rugby-related concussion (yes/no)	How many times have you been concussed whilst playing or training for rugby? (n=101)	No. of concussions (rugby and non-rugby)? (n=9)	Have you ever been dazed (‘dinged’) during a match?+Have you ever been unconscious (‘knocked out’) during a match? (n=14)	Have you ever been dazed (‘dinged’) during a match?+Have you ever been unconscious (‘knocked out’) during a match? (n=84^1^)
*Rugby-related (dichotomous)*				
Ever suffered a rugby-related concussion with loss of consciousness (yes/no)	How many times have you been concussed whilst playing or training for rugby?+Temporary loss of consciousness (n=101)	-	Have you ever been unconscious (‘knocked out’) during a match? (n=14)	Have you ever been unconscious (‘knocked out’) during a match? (n=86^1^)
*Rugby-related with LOC (dichotomous)*				
Ever suffered any concussion (yes/no)	How many times have you been concussed whilst playing or training for rugby?+How many times have you been concussed when you have not been playing or training for rugby? (n=101)	How many times have you been concussed whilst playing or training for rugby?+How many times have you been concussed when you have not been playing or training for rugby? (n=9)	-	How many times have you been concussed? Please include all sporting and non-sporting concussions (concussed/don’t know) (n=67)
*Any concussion (dichotomous)*				
No. of any concussions^2^	How many times have you been concussed whilst playing or training for rugby?+How many times have you been concussed when you have not been playing or training for rugby? (n=83, ever concussed only)	How many times have you been concussed whilst playing or training for rugby?+How many times have you been concussed when you have not been playing or training for rugby? (n=9)	-	How many times have you been concussed? Please include all sporting and non-sporting concussions (n=53, ever concussed only)
*Any concussion (numerical)*				

BRAIN, BRain health and healthy AgeINg in retired rugby union players; BRAIN-Q, BRAIN questionnaire tool; tBRAIN-Q, telefornic version of the BRAIN-Q; MSK study, Oxford and Cambridge University players and English international rugby players; LOC, loss of consciousness.

1 Number differs from total allowing for “don’t know” answers and missing values.

2 Of those that answered “yes” to “ever concussed.”

**Table 2.** Concussion definitions provided by each assessment tool

MSK-Q (main)	BRAIN-Q
Concussion is defined as an injury resulting from a blow to the head that caused an alteration in metal status and one or more of the following symptoms: headache, nausea, vomiting, dizziness/balance problems, fatigue, trouble sleeping, drowsiness, sensitivity to light or noise, blurred vision, difficulty remembering and difficulty concentrating	Concussion is defined as an alteration in brain function, caused by an external force; Symptoms include: a decreased level/loss of consciousness; Memory loss (before or after the injury); Weakness/temporary Paralysis; Loss of balance; Change in vision (e.g., blurriness, double vision); Co-ordination difficulties; Numbness; Decreased sense of smell; Difficulty understanding what others are saying; Difficulty communicating with others; Confusion, disorientation, or slowed thinking
	Loss of consciousness is not required for a concussion to be diagnosed

MSK-Q, muscle skeletal questionnaire; BRAIN, BRain health and healthy AgeINg in retired rugby union players; BRAIN-Q, BRAIN questionnaire tool.

**Table 3.** Cross-tabulation of the dichotomous rugby-related concussion^1^

Variables	BRAIN-Q		
	Yes	No	Total
Ever concussed (rugby-related)			
MSK-Q – main+pilot			
Yes	72	6	78
No	7	13	20
Total	79	19	98
Agreement=86.7% (κ=0.6; 95% CI, 0.4, 0.8)			
Ever concussed with loss of consciousness (rugby-related)			
MSK study – main+pilot			
Yes	46	7	53
No	11	36	47
Total	57	43	100
Agreement=82.0% (κ=0.6; 95% CI, 0.5, 0.8)			
Ever concussed (any)			
MSK study – main only			
Yes	51	2	53
No	6	8	14
Total	57	10	67
Agreement=88.1% (κ=0.6; 95% CI, 0.4, 0.9)			

BRAIN, BRain health and healthy AgeINg in retired rugby union players; BRAIN-Q, BRAIN questionnaire tool; MSK-Q, muscle skeletal questionnaire; CI, confidence interval; MSK study, Oxford and Cam bridge University players and English international rugby players.

1 Variable assessed with BRAIN-Q and MSK-Q; of the dichotomous rugby-related concussion with loss of consciousness (yes/no) variable assessed with the BRAIN-Q and MSK-Q (MSK variable definition 2); and of the dichotomous any concussion (yes/no) variable assessed with the BRAIN-Q and MSK-Q.

**Table 4.** Total number of rugby and non-rugby-related concussions reported through the BRAIN-Q (initial assessment) and tBRAIN Recall (follow-up phone call)

Participant	BRAIN-Q			tBRAIN-Q Recall			Difference in total no. of concussions
	Rugby-related concussions	Non-ruby-related concussions	Total no. of concussions	Rugby-related concussions	Non-rugby-related concussions	Total no. of concussions	
A	1	1	2	1	1	2	0
B	2	1	3	2	1	3	0
C	2	0	2	2	0	2	0
D	1	0	1	2	0	2	1
E	3	0	3	3	0	3	0
F	1	0	1	1	0	1	0
G	2	0	2	2	1	3	1
I	1	0	1	1	0	1	0
J	9	1	10	9	0	9	1
Concordance statistic							
Rugby-related concussions: 0.990 (0.975, 1.004)							
Non-rugby related concussions: 0.500 (-0.056, 1.056)							
Total no. of concussions: 0.973 (0.943, 1.003)							

BRAIN, BRain health and healthy AgeINg in retired rugby union players; BRAIN-Q, BRAIN questionnaire tool; tBRAIN-Q, telefornic version of the BRAIN-Q.

## SUPPLEMENTARY MATERIALS

Korean version is available at https://www.e-epih.org/.

Supplementary Material 1. The BRAIN Study Concussion Questionnaire BRAIN-Q
