# Supplementary material for: The BRAIN-Q, a tool for assessing self-reported sport-related concussions for epidemiological studies
Source: Epidemiol Health. 2021 Oct 19;43:e2021086. doi: 10.4178/epih.e2021086 (PMC8863616; doi:10.4178/epih.e2021086)
Supplement: Supplementary Material 1. — The BRAIN Study Concussion Questionnaire BRAIN-Q [file epih-43-e2021086-suppl.docx]

# Supplementary Material 1.

The BRAIN Study Concussion Questionnaire

BRAIN-Q

| **Concussion Questionnaire** | | |
| --- | --- | --- |
| *Please read aloud the following definition of concussion:*  ***Concussion is defined as an alteration in brain function, caused by an external force. Symptoms include:***   - ***A decreased level / loss of consciousness*** - ***Memory Loss (before or after the injury)*** - ***Weakness / Temporary Paralysis*** - ***Loss of balance*** - ***Change in vision (e.g. blurriness, double vision)*** - ***Co-ordination difficulties*** - ***Numbness*** - ***Decreased sense of smell*** - ***Difficulty understanding what others are saying*** - ***Difficulty communicating with others*** - ***Confusion, disorientation, or slowed thinking***   **Loss of consciousness is not required for a concussion to be diagnosed.**  *Do you understand this definition?* | | |
| 1. | *How many times have you been concussed whilst playing or training for Rugby?* | __________ |
| 2. | *How many times have you been concussed when you have not been playing or training for rugby?* | __________ |

*With the help of your assessor please complete this timeline to indicate when experienced a concussion.*

*Please indicate (1) the year in which the concussion occurred, and (2) whether the concussion was experienced whilst playing or training for rugby.*

*To aid your recall you may wish to mark on the timeline key periods or events within your life, such as date of birth, time spent in education and years playing rugby. Please see an example below, and complete your own timeline on the following page.*

**Example**
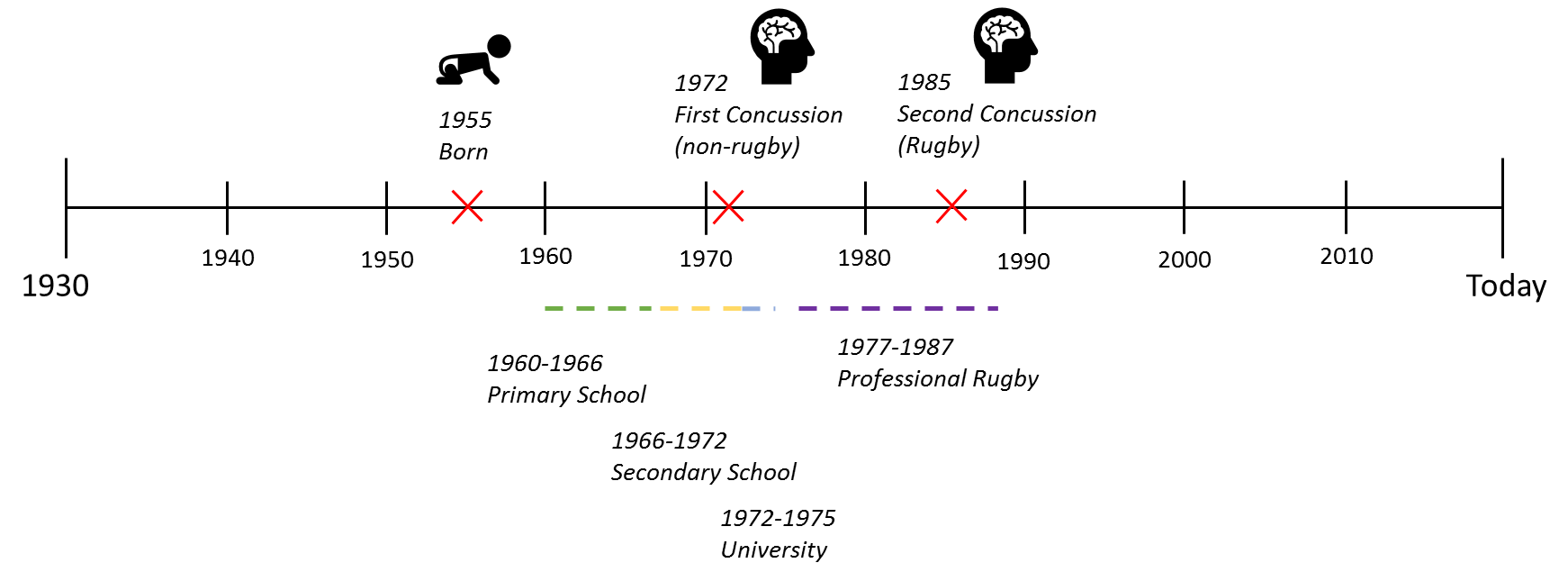


**Your timeline**

**
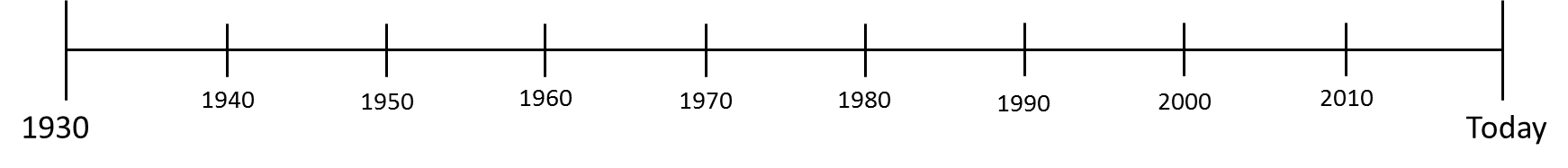
**

*In chronological order using your timeline, please complete the following details for each episode of concussion. For each episode, please select all that apply and fill the gaps. If you are unsure, please do not tick the box.*

| First Concussion | | |
| --- | --- | --- |
| I was ___________ years old  I reported a fracture of the skull or any other head bone  I was admitted in hospital for _____ days  I was seen in A&E but not admitted overnight  I experienced temporary loss of consciousness | | □  □  □  □ |
| What were you doing when you had your first concussion? | □ playing/training for Rugby  □ playing/training for another sport  (please specify _________________________)  □ motor vehicle accident (car, motorbike, bus)  □ other (please specify ______________________) | |
| Second Concussion | | |
| I was ___________ years old  I reported a fracture of the skull or any other head bone  I was admitted in hospital for _____ days  I was seen in A&E but not admitted overnight  I experienced temporary loss of consciousness | | □  □  □  □ |
| What were you doing when you had your second concussion? | □ playing/training for Rugby  □ playing/training for another sport  (please specify _________________________)  □ motor vehicle accident (car, motorbike, bus)  □ other (please specify ______________________) | |
| Third Concussion | | |
| I was ___________ years old  I reported a fracture of the skull or any other head bone  I was admitted in hospital for _____ days  I was seen in A&E but not admitted overnight  I experienced temporary loss of consciousness | | □  □  □  □ |
| What were you doing when you had your third concussion? | □ playing/training for Rugby  □ playing/training for another sport  (please specify _________________________)  □ motor vehicle accident (car, motorbike, bus)  □ other (please specify ______________________) | |

| Fourth Concussion | | |
| --- | --- | --- |
| I was ___________ years old  I reported a fracture of the skull or any other head bone  I was admitted in hospital for _____ days  I was seen in A&E but not admitted overnight  I experienced temporary loss of consciousness | | □  □  □  □ |
| What were you doing when you had your fourth concussion? | □ playing/training for Rugby  □ playing/training for another sport  (please specify _________________________)  □ motor vehicle accident (car, motorbike, bus)  □ other (please specify ______________________) | |
| Fifth Concussion | | |
| I was ___________ years old  I reported a fracture of the skull or any other head bone  I was admitted in hospital for _____ days  I was seen in A&E but not admitted overnight  I experienced temporary loss of consciousness | | □  □  □  □ |

| What were you doing when you had your fifth concussion? | □ playing/training for Rugby  □ playing/training for another sport  (please specify _________________________)  □ motor vehicle accident (car, motorbike, bus)  □ other (please specify ______________________) |
| --- | --- |
| *Please let your assessor know if you have experienced more than five concussions and they will provide you with further copies of the questionnaire.* | |
